# Prioritizing clinical data for psychiatric inpatient dashboards: insights from a nationwide survey of German university centers

**DOI:** 10.3389/fdgth.2025.1617116

**Published:** 2026-01-09

**Authors:** Julian Herpertz, Alina Brockmann, Maike Richter, Rogério Blitz, Marius Gruber, Kira F. Ahrens, Paula Rehm, Ramona Leenings, Luise Victoria Claaß, Jonathan Repple, Nils Opel

**Affiliations:** 1 Department of Psychiatry & Neuroscience, Charité Berlin University Medicine, Campus Benjamin Franklin, Berlin, Germany; 2 Department of Psychiatry and Psychotherapy, Jena University Hospital, Jena, Germany; 3 German Center for Mental Health (DZPG), partner site Berlin-Potsdam, Berlin, Germany; 4 Institute for Translational Psychiatry, University of Münster, Münster, Germany; 5 Department of Psychiatry, Psychosomatic Medicine and Psychotherapy, Goethe University Frankfurt, University Hospital, Frankfurt, Germany; 6 Cooperative Brain Imaging Center—CoBIC, Goethe University Frankfurt, Frankfurt, Germany; 7 Center for Intervention and Research on Adaptive and Maladaptive Brain Circuits Underlying Mental Health, Jena-Magdeburg-Halle, Germany

**Keywords:** affective disorder, digital dashboard, digital psychiatry, inpatient psychiatry, survey study, mental health, digital mental health

## Abstract

**Introduction:**

As digital data collection becomes increasingly integrated into the treatment of patients with affective disorders, the use of dashboards to visualize this information for clinicians is gaining importance. However, the question of which parameters should be prioritized for display remains largely unaddressed. This study aims to identify the parameters that physicians working in psychiatric facilities consider most important for inclusion in dashboard infrastructures supporting the inpatient care of patients with affective disorders.

**Methods:**

From July 2024 to August 2024, we conducted a survey among 57 physicians working in psychiatric facilities at German university centers with varying levels of experience. We asked them to rank the relevance of 22 pre-specified key clinical parameters for digital dashboard displays. Additionally, we assessed whether characteristics such as gender, age, years of professional experience, and professional seniority influenced these preferences.

**Results:**

Forty-six physicians (80%) physicians completed the data entry. Across the sample, current suicidality emerged as the most important parameter to clinicians. Other highly ranked parameters included information on previous pharmacological antidepressant treatment attempts and data on the course of disease such as year of onset and the number of episodes. The influence of clinician-related factors on parameter prioritization was limited, supporting the generalizability of the findings.

**Discussion:**

Our findings provide practical guidance for the refinement of digital dashboards tailored to the clinical needs in the treatment of affective disorders. Future research should incorporate the perspectives of the entire multidisciplinary care team and evaluate the feasibility and clinical integration of such dashboards to ensure their broader applicability and effectiveness in routine practice.

## Introduction

An inpatient symptom monitoring system in psychiatry offers the potential to accurately track depressive symptom trajectories throughout the course of treatment. Especially when data is directly integrated into the electronic health records of patients, it allows for the early detection of behavioral anomalies and the identification of individuals at increased risk for symptom exacerbation (1). Since symptom severity in psychiatric populations cannot only be reliably assessed through external observations alone, and such assessments would require substantial clinical resources, digital self-reported data collections present a feasible alternative (2, 3).

Digital dashboards play an integral role in harnessing the full potential of the gathered clinical data (4). They can be defined as digital interfaces that aggregate, analyze, and visualize patient data and therefore help to transform complex information into actionable insights. In modern health systems, they are widely viewed as indispensable components of data-informed care (5). When integrating a dashboard into clinical workflows, a critical first step is to decide on a limited number of the most relevant parameters to present. This is because of two central reasons. First, to enhance clinical usability: Dashboards should provide a concise overview that saves clinicians time and effort, enabling them to access essential patient insights at a glance, rather than having to manually retrieve and interpret scattered data (6). Therefore, too many data points would compromise clarity and thereby reduce clinical utility. Second, to uphold patient engagement in the regular data collection, as excessive or burdensome assessments may lead to disengagement over time (7).

Several digital dashboards for mental health care have been developed and evaluated in research and pilot implementation settings, and prior studies have shown that both clinicians and patients recognize their potential benefits for clinical decision-making and treatment monitoring (8–11). However, most of these systems remain either experimental or locally implemented and have not yet been adopted into routine inpatient psychiatric care, particularly within the German healthcare system. Moreover, much of the existing literature focuses on technical feasibility or general usability rather than on determining which clinical information clinicians actually consider most relevant for display. As a result, dashboard designs are often guided by what data are available rather than by what data are clinically meaningful, and usability evaluations frequently rely on generic questionnaires that are not grounded in clinical workflow needs (12).

Critically, the success of digital dashboards depends not only on their technological robustness or visual design, but also on the clinical salience of the data they present. If the displayed parameters do not reflect the actual informational needs of clinicians, dashboards risk being underutilized or misaligned with decision-making priorities. While the importance of user-centered design in dashboard development has been widely acknowledged (5, 13), to our knowledge, no systematic efforts have been made to empirically determine which specific clinical parameters psychiatrists deem most relevant to include.

This cross-sectional study aims to fill that gap. Rather than beginning with technology implementation, we adopted a formative, clinician-centered approach as a necessary first step toward effective dashboard design. In order to empirically ground future design, implementation, and evaluation efforts, we systematically evaluated clinician preferences across multiple German university medical centers. Specifically, physicians involved in the treatment of patients with affective disorders were presented with a selection of routinely used clinical parameters and asked to rank them based on their subjective assessment of importance. The primary objective was to identify which types of clinical information are considered most relevant for supporting day-to-day clinical decision-making, thereby informing the selection of content to be featured in a digital dashboard.

We hypothesized that clinicians would show a high degree of agreement regarding the most important parameters for inclusion, with relatively little ambiguity in identifying the variables deemed most clinically relevant. However, we also expected that the ranking of parameters would be influenced by clinicians’ level of professional experience.

## Methods

On July 7th, 2024 a total of 57 psychiatrists working in psychiatric institutions at German university centers were invited via email to participate in a web-based survey hosted on LimeSurvey (14). Inclusion criteria for participation in the survey were: current employment as a psychiatrist in a clinical setting treating affective disorders at a German university center, a minimum of five years of professional experience, board certification in psychiatry, and sufficient proficiency in the German language. Eligible psychiatrists were identified through hospital websites and professional psychiatric society directories and were invited via email, which included a brief description of the study. The sample included physicians from various regions across Germany and represented different stages of clinical careers.

A reminder email was sent four weeks after the initial invitation and the data collection ended six weeks after the first invitation. The questionnaire collected information on participants’ sociodemographic characteristics and clinical experience. In addition, clinicians were asked to rank 22 predefined parameters according to their perceived importance for inclusion in a digital dashboard intended for use in inpatient care of patients with affective disorders. Ranking was facilitated using a drag-and-drop functionality in a descending order.

The parameters were predefined based on a pragmatic expert consensus among three professionals with experience in psychiatry and digital mental health. Variables that are routinely documented in the electronic health record, such as age, gender and current medication, were excluded from the list, as the focus was on parameters that could provide additional clinical value when visualized in a digital dashboard.

We conducted a ranking analysis to assess the relative importance of parameters as perceived by clinicians. For each parameter, we computed the mean rank by averaging the rank values across all respondents.

To explore whether clinician characteristics influenced the prioritization of parameters proposed for inclusion, we conducted a series of regression analyses. We performed separate ordinary least squares regressions for each of the 22 parameters, predicting the rank a clinician assigned to that parameter using one predictor at a time. Gender, the age of clinicians, their years of experience and their position on the physicians’ career hierarchy (resident, attending physician, head of department, senior physician) served as predictors.

After completing the drag-and-drop ranking task, clinicians were invited to provide open-ended suggestions regarding additional information, beyond the predefined 22 parameters, that they believed could be valuable for inclusion in a digital dashboard. We report all additional parameters that were suggested by at least two different clinicians.

## Results

Of 57 clinicians that were contacted for the data collection, 46 (80.07%) completed the questionnaire. All respondents were physicians in psychiatry working at German university clinics, except for one who reported working at a specialized clinic and was therefore excluded from further analyses. The mean age of included participants was 47.13 (SD. 9.02) years and the mean time of clinical practice was 19.43 (SD. 8.56) years. Most participants identified as senior physicians (*n* = 32; 71.11%), followed by heads of department (*n* = 9; 20.00%), attending physicians (*n* = 3; 6.67%), and one resident (*n* = 1; 2.22%). The majority of participating clinicians was male (*n* = 33; 73.33%) and 12 participants were female (*n* = 12; 26.67%).

**Figure 1:**
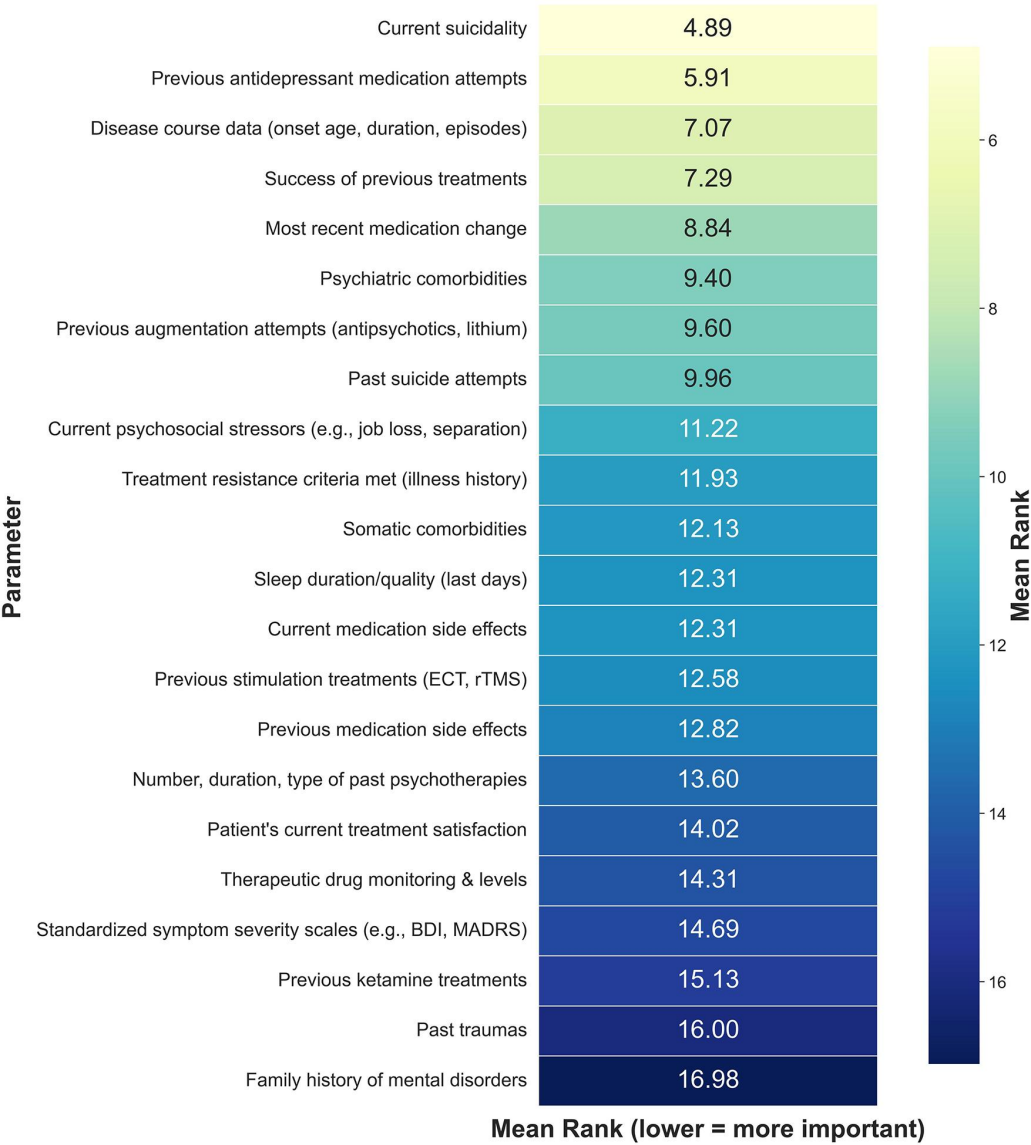
Mean ranking of clinical parameters by perceived importance for inclusion in a digital psychiatric dashboard. Lower mean values indicate higher perceived importance. Each parameter was ranked by 45 clinicians from 1 (most important) to 22 (least important). The heatmap displays the mean rank across all respondents.

Clinicians were asked to rank 22 clinical parameters based on their perceived importance for inclusion in a digital dashboard designed to support inpatient care of patients with an affective disorder. Current suicidality was rated as the most important parameter (mean rank = 4.89), followed by information on previous pharmacological antidepressant treatment attempts (5.91) and data on the course of disease such as the year of onset and the number of episodes (7.07). At the lower end of the ranking spectrum was information on whether patients were exposed to trauma (16.00) and if other family members suffered from a mental disorder (Family history of mental disorder; 16.98; Figure 1).

**Figure 2:**
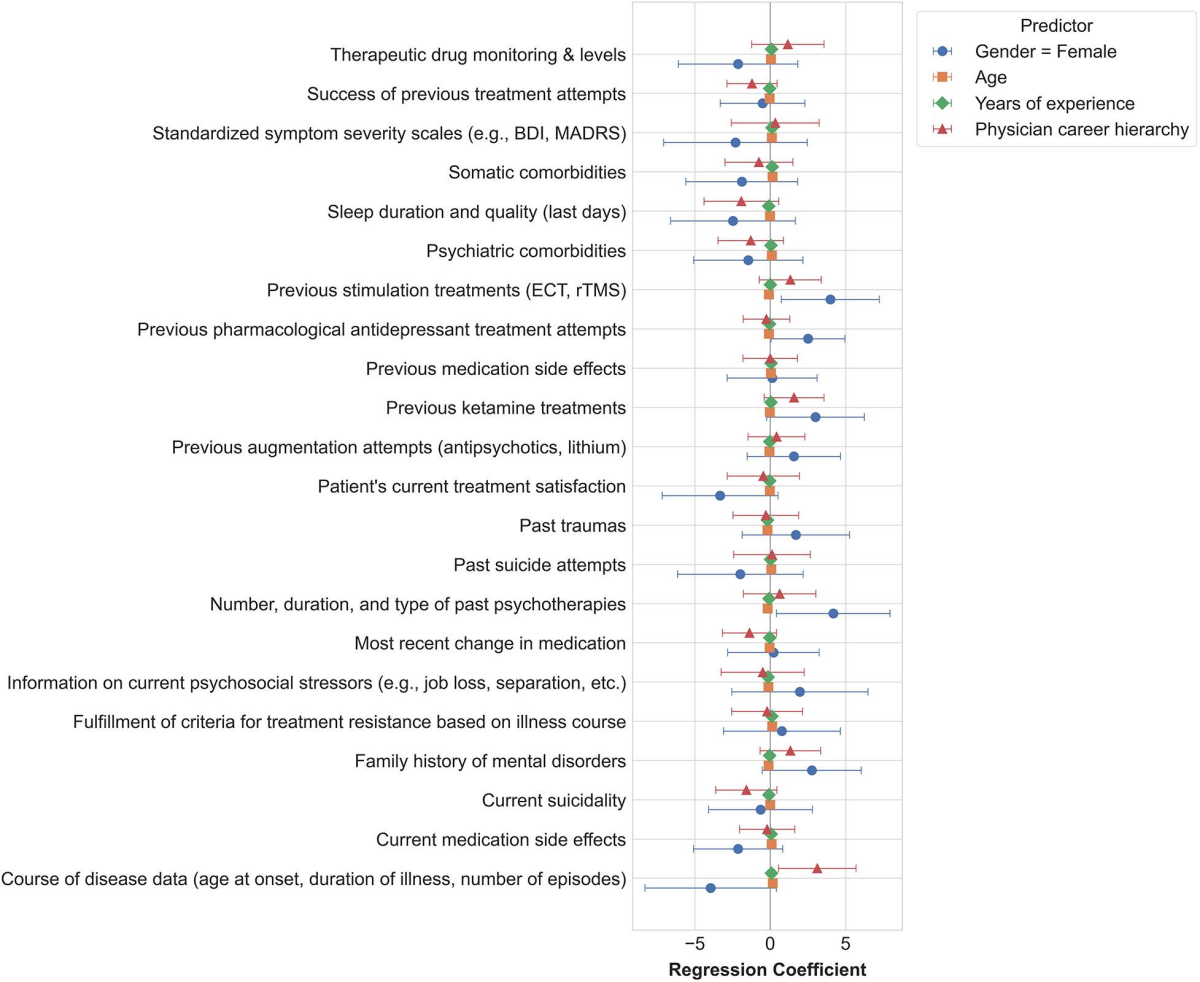
Univariate regression coefficients (with 95% confidence intervals) for each clinical parameter predicted by clinician characteristics.

Across all respondents, there was a high degree of agreement regarding which clinical parameters should be prioritized for inclusion in digital dashboards. This consistency was observed largely independent of clinicians’ individual characteristics such as sex, age, clinical experience, or hierarchical position. Nonetheless, a few associations between clinician characteristics and the prioritization of specific parameters emerged. Female clinicians were significantly less likely to assign high importance to data on previous stimulation treatments (e.g., ECT, rTMS; *β* = 3.98, *p* = .021), and the number, duration, and type of previously applied psychotherapeutic interventions (*β* = 4.18, *p* = .035). Additionally, a clinician’s career stage was significantly associated with the perceived importance of course of disease data, with more senior physicians tending to rate this parameter as less important (*β* = 3.13, *p* = .022). Older clinicians, in turn, were more likely to emphasize the importance of patient experiences with past trauma (*β* = −1.87, *p* = .036; Figure 2).

A positive coefficient indicates that an increase in the predictor is associated with lower importance, while a negative coefficient indicates an association with higher importance.

When analyzing the additional parameters suggested by clinicians, we found that three respondents expressed a desire for more detailed information on patients’ social support, for example, whether family members were available to provide support. Additionally, clinicians indicated interest in more comprehensive data on patients’ current life situation, including whether they lived independently, with family, in assisted living arrangements, or whether they were currently employed. Moreover, two clinicians respectively suggested including information on personality measures, a history of psychotic symptoms, and more detailed data on current inpatient medication, preferably presented with a temporal overview.

## Discussion

In our study, we investigated which parameters clinicians consider most important for inclusion on a digital dashboard aimed at improving the inpatient care of patients with affective disorders. The clinical utility of these prioritized parameters extends beyond physicians, supporting informed decision-making across the entire multidisciplinary team, including nursing staff, psychologists and social workers. Capitalizing on the rapid accessibility of digital dashboards, this information should be available to all team members empowered to intervene, ensuring timely responses and enhanced safety. Our findings contribute to the foundational stages of dashboard development, where clinician input is crucial to ensure relevance and clinical utility. Although usability and outcome data are not yet available, incorporating clinician priorities at the outset aligns with user-centered design principles and addresses a common gap in the development of digital health tools. Certain parameters consistently emerged as particularly important across respondents. Our findings can inform policymakers, clinical leaders, and, most importantly, dashboard developers in selecting which variables to prioritize.

To date, only a limited number of studies have systematically explored the key characteristics that digital dashboards in mental health should exhibit, let alone the parameters they should assess. Chang et al. (15) and Scheuer and Torous (16) emphasize that dashboards and their visualizations should be highly intuitive and easy to comprehend, ensuring they can serve not only as a tool for clinicians but also as a source of guidance for patients. Discussing dashboard results collaboratively with patients can enhance trust between clinicians and patients, while also strengthening motivation and fostering a sense of validation in patients. While the general expansion of collaborative analysis of patient data visualizations is widely supported, potential negative effects have also been noted. In particular, patients who show limited progress during inpatient treatment may experience frustration or even a nocebo effect when confronted with stagnant or unfavorable results (15, 17).

For dashboard developers, a practical implication of our results could therefore be to display a parameter such as sleep quality and its recent trajectory (ranked as moderately important) in a clear and easily interpretable format. This would enable clinicians to review the data together with their patients, providing an opportunity for patients to reflect on their recent sleep patterns and consider potential contributing factors. A highly relevant use case is clinical ward rounds, during which clinicians must rapidly grasp a patient's status and recent clinical trajectory. Current suicidality, being the most relevant parameter to clinicians, should equally be displayed in an easily interpretable manner, for example, using a traffic light system that immediately informs clinicians about recent developments and changes (light switches red).

For clinicians, the key takeaway is to allocate time to discuss these developments with their patients, thereby fostering engagement, insight, and shared understanding. Recognizing that clinicians may not always have the capacity to engage in detailed discussions of visualized data with patients, the concept of ‘digital navigators’ has gained considerable attention. These professionals are specifically trained to interpret digital health information and facilitate meaningful conversations with psychiatric patients (18, 19).

In a 2014 online survey, 784 experts in psychiatry provided feedback on which initial assessment parameters they considered essential for improving treatment outcomes (20). The scope of assessment topics in the survey was considerably broader than the parameters proposed in our study. However, suicide risk and information on psychiatric treatment history received the highest levels of agreement, closely aligning with our own findings. This suggests that, more than a decade later, and regardless of the digital focus of our research, the specific psychiatric disorder under investigation, or the country in which the study was conducted, the core parameters deemed most relevant by clinicians in psychiatry have remained largely consistent. The American Psychiatric Association derived clinical guidelines from the 2014 survey, and while many of these recommendations are already reflected in the parameters most frequently endorsed in our clinician survey, they also suggest additional areas of assessment that we had not yet considered for inclusion in the dashboard (21). Information on substance use history and prior aggressive behavior ranked highly among the recommended assessment domains and should therefore be considered for inclusion in a digital dashboard. However, while these parameters are of general relevance in psychiatric assessment, they are less specific to affective disorders, the primary focus of the present study (22, 23).

The lower prioritization of historical parameters, such as past traumas and family history, likely reflects the specific utility of digital dashboards as tools for monitoring dynamic changes rather than reviewing static biographical data. While these factors are fundamental to case formulation, they remain constant throughout the inpatient stay and do not require the real-time visualization that characterizes dashboard functionality.

Our findings indicate that female clinicians tend to place less emphasis on information about previous neurostimulation interventions, antidepressant pharmacotherapy, and detailed accounts of prior psychotherapeutic treatments compared to their male counterparts. Studies on gender differences within clinical settings are scarce and have predominantly focused on differences in emotional processing when interacting with patients (24, 25). However, given the distinct imbalance in gender distribution within our sample (only 12 female clinicians), the interpretation of these subgroup differences is limited. Future survey studies should aim to include a larger number of female clinicians to determine whether these findings can be replicated. The finding that gender, age, years of experience, and position on the medical career ladder had only limited influence on the ranking of parameter importance supports the generalizability of our results, indicating that they are largely independent of clinician-related factors.

Several limitations must be acknowledged. A key limitation of our study lies in the restricted scope of participants. We surveyed only physicians, primarily experienced clinicians working at German university hospitals, thus excluding the broader multidisciplinary teams involved in psychiatric care. While we posit that the prioritized parameters have cross-disciplinary utility, the specific perspectives of nursing staff, psychologists, and social workers were not empirically captured. Without their perspectives, the generalizability and ecological validity of our findings are limited. Our sample was limited not only in terms of professional roles but also regarding the clinical contexts covered. It remains an open question whether the prioritization of parameters identified here would differ in outpatient settings, across distinct diagnostic groups (e.g., psychotic or substance use disorders), or in the context of long-term treatment planning. As our survey focused on inpatient care without distinguishing between acute vs. long-term trajectories, future studies should explore how parameter relevance may shift across care contexts, diagnoses and treatment phases.

Furthermore, although our survey identified which parameters clinicians find most relevant, we did not assess how these data should be visually presented or integrated into clinical workflows. Dashboard usability depends not only on content but also on interaction design. While we included a free-text field for additional suggestions, future research should complement our approach with qualitative or mixed-methods designs to explore why particular parameters matter and how they should be implemented in practice.

When translating our findings into dashboard design, a key challenge lies in balancing information richness with clarity. Displaying too many data points at once may undermine decision-making efficiency. Our ranking results directly inform this balance by identifying which parameters clinicians consider most essential for rapid clinical orientation. In addition to usability considerations, ethical and data protection issues must be carefully addressed when developing dashboards in psychiatric care. Many of the parameters prioritized by clinicians are highly sensitive. Thoughtful dashboard design should therefore not only prioritize clinical relevance but also ensure privacy-respecting data handling.

## Conclusion

Digital data collection and the visualization of results via digital dashboards will likely become indispensable in mental health care, not only in outpatient settings but also in inpatient psychiatry. The present study therefore serves as initial guidance in identifying which parameters are of greatest value to clinicians for inclusion in dashboard infrastructures within mental healthcare. Building on these findings, the next step is the translation of the prioritized parameters into a dashboard prototype. Future research should focus on the pilot integration within a real-world inpatient setting, specifically evaluating its usability, impact on time-efficiency, and acceptance by the multidisciplinary team.

## Data Availability

The raw data supporting the conclusions of this article will be made available by the authors, without undue reservation.
